# Long-term experience with triheptanoin in 12 Austrian patients with long-chain fatty acid oxidation disorders

**DOI:** 10.1186/s13023-020-01635-x

**Published:** 2021-01-14

**Authors:** Thomas Zöggeler, Katharina Stock, Monika Jörg-Streller, Johannes Spenger, Vassiliki Konstantopoulou, Miriam Hufgard-Leitner, Sabine Scholl-Bürgi, Daniela Karall

**Affiliations:** 1 Department of Pediatrics I (Inherited Metabolic Disorders), Medical University of Innsbruck, Anichstrasse 35, 6020 Innsbruck, Austria; 2 Department of Pediatrics III (Cardiology), Medical University of Innsbruck, Innsbruck, Austria; 3 University Children’s Hospital, Salzburger Landeskliniken (SALK) and Paracelsus Medical University (PMU), Salzburg, Austria; 4 Department of Pediatrics and Adolescent Medicine, Medical University of Vienna, Vienna, Austria; 5 Department of Internal Medicine III (Clinical Division of Endocrinology and Inherited Metabolic Disorders), Medical University of Vienna, Vienna, Austria

**Keywords:** Inherited metabolic disorders, Inborn errors of metabolism, Anaplerotic effect, Fat intake regimen, Long-chain ß-oxidation disorders

## Abstract

**Background:**

Long-chain fatty acid oxidation disorders (LC-FAOD) are a group of rare inborn errors of metabolism with autosomal recessive inheritance that may cause life-threatening events. Treatment with triheptanoin, a synthetic seven-carbon fatty acid triglyceride compound with an anaplerotic effect, seems beneficial, but clinical experience is limited. We report our long-term experience in an Austrian cohort of LC-FAOD patients.

**Methods:**

We retrospectively assessed clinical outcome and total hospitalization days per year before and after start with triheptanoin by reviewing medical records of 12 Austrian LC-FAOD patients

**Results:**

For 12 Austrian LC-FAOD patients at three metabolic centers, triheptanoin was started shortly after birth in 3/12, and between 7.34 and 353.3 (median 44.5; mean 81.1) months of age in 9/12 patients. For 11 pediatric patients, mean duration of triheptanoin intake was 5.3 (median 3.9, range 1.2–15.7) years, 10/11 pediatric patients have an ongoing intake of triheptanoin. One patient quit therapy due to reported side effects. Total hospitalization days per year compared to before triheptanoin treatment decreased by 82.3% from 27.1 (range 11–65) days per year to 4.8 (range 0–13) days per year, and hospitalization days in the one year pre- compared to the one year post-triheptanoin decreased by 69.8% from 27.1 (range 4–75) days to 8.2 (range 0–25) days. All patients are in good clinical condition, show normal psychomotor development and no impairment in daily life activities.

**Conclusion:**

In this retrospective observational study in an Austrian LC-FAOD cohort, triheptanoin data show improvement in disease course. Triheptanoin appears to be a safe and beneficial treatment option in LC-FAOD. For further clarification, additional prospective randomized controlled trials are needed.

## What is known?

- Long-chain fatty acid oxidation disorders are rare, but may cause life-threatening events and long-term neurological impairment.
- Clinical outcome is variable.
- Triheptanoin, a synthetic seven odd-chain fatty acid triglyceride was recently approved by the US FDA in summer 2020. Approval for FAODs in Europe is pending.

## What is new?

- Daily treatment with 0.5–1.0 g/kg/day triheptanoin while allowing a total fat intake of up to 30% of total daily energy shows good long-term clinical outcome in patients with LC-FAOD.
- Maintaining patients in an anabolic state is crucial and outweighs the effect of stringent fat restriction.

## Introduction

**Fig. 1:**
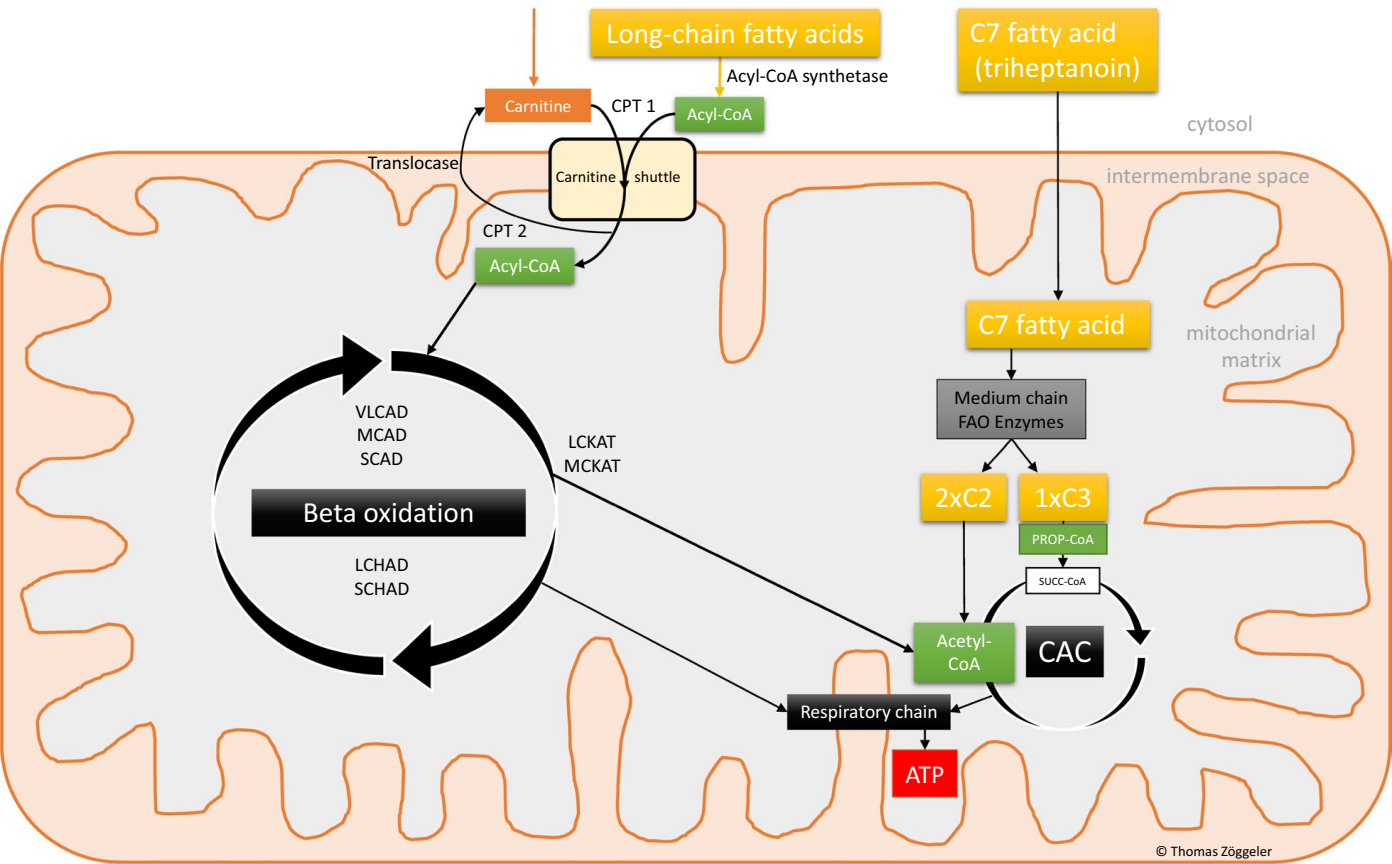
Diagram of the mitochondrial metabolic LC-FAOD and C7 pathways. Abbreviations: CPT1: carnitine palmitoyltransferase 1; CPT2: carnitine palmitoyltransferase 2; VLCAD: very long-chain acyl-CoA dehydrogenase; MCAD: medium-chain acyl-CoA dehydrogenase; SCAD: short-chain acyl-CoA dehydrogenase; LCHAD: long-chain 3-hydroxyacyl-CoA dehydrogenase; SCHAD: short-chain 3-hydroxylacyl-CoA dehydrogenase; LCKAT: long-chain 3-ketothiolase; MCKAT: medium-chain 3-ketoacyl-CoA thiolase; PROP-CoA: propionyl-CoA; FAO: fatty acid oxidation; CAC: citric acid cycle; ATP: adenosine triphosphate. Copyright Thomas Zöggeler MD

Long-chain fatty acid oxidation disorders (LC-FAOD) are a group of rare inherited metabolic disorders with autosomal recessive inheritance with defects in the mitochondrial long-chain fatty acid oxidation pathway (Fig. 1) [1, 2]. Disruptions in the pathway cause mitochondrial energy deficiency and lead to a toxic accumulation of metabolic intermediates [3]. Patients with LC-FAODs are therefore prone to life-threatening episodes, especially during periods of fasting, fever and physical stress. Clinical presentations include short-term symptoms such as hypoglycemia, acidosis, rhabdomyolysis and liver dysfunction, as well as long-term complications such as (cardio-)myopathy, retinopathy or polyneuropathy [3]. The current gold standard for clinical treatment is dietary management with fat-defined, isocaloric nutrition, regular food intake and supplementation of medium-chain triglycerides (MCTs) to establish anabolism [4]. Despite these measures, morbidity and mortality remain high [5]. With no approved specific medication for treatment of LC-FAODs available, triheptanoin constitutes a promising agent that is currently used in compassionate care programs [6, 7]. Triheptanoin is a highly purified, synthetic seven odd-chain fatty acid triglyceride that can bypass the deficient long-chain oxidation. The drug is metabolized to propionyl-CoA and acetyl-CoA, both essential precursors for the citric acid cycle (CAC) (Fig. 1). Propionyl-CoA is metabolized to succinyl-CoA, which resupplies the CAC intermediates, providing a more effective production of adenosine triphosphate (ATP) in patients with LC-FAOD [8]. This mechanism potentially increases gluconeogenesis and glycogen stores [9]. Owing to the rareness of LC-FAODs, data on long-term outcome of patients with LC-FAOD on triheptanoin are still scarce. However, a few studies have yielded promising results [2, 6, 7, 10, 11].

We here report our long-term experience with triheptanoin in 12 Austrian patients with LC-FAOD.

## Patients and methods

### Study population and main outcome parameter

We retrospectively collected data from 12 patients with LC-FAOD from three Austrian metabolic centers by reviewing their medical history. Nine patients were cared for in Innsbruck, one in Salzburg (Patient 10) and one in Vienna (Patient 11); outcome of one additional patient in Vienna (Patient 12) is reported separately. Data obtained from medical records cover the patients’ history until May 2020. Epidemiological data include sex, age, diagnosis, onset of disease, time to diagnosis, family history, newborn screening results, symptoms, dietary management, and initiation of triheptanoin treatment. Furthermore, number of hospital admissions (in days per year), number of episodes of rhabdomyolysis (defined as peak creatine kinase concentration above 500 U/I), as well as concomitant short- and long-term complications (cardiomyopathy, hepatopathy and retinopathy) were assessed. Total fat intake and quality of fats was obtained from dietary protocols. Additionally, late night feeds and nasogastric or PEG-tube feeding were assessed. Neurological outcome was documented according to self-reported school performance and need for physical support (i.e. wheelchair-bound). Weight, height and BMI percentiles were calculated according to reference data sets [12, 13]. Main outcome parameter was total hospitalization days per year before and after treatment with triheptanoin.

### Patients’ characteristics (Table 1)

**Table 1:** Underlying LC-FAOD diagnosis, organ involvement and clinical outcome in 12 Austrian LC-FAOD patients

Patient	Sex	Current age (years)	LC-FAOD diagnosis	Age at diagnosis (months)	Clinical symptoms (decompensation) at diagnosis	Newborn screening	Age at start of C7 therapy (years)	Hepatopathy^2^		Cardiomyopathy^3^		Retinopathy		NG or PEG tube	Special-needs school	Poly-neuropathy
								At age (months/years)		At age (months/years)		At age (months/years)				
1	Male	20.5	LCHADD	23.1	Yes	Not yet established^1^	4.8	Yes	23/1.9	Yes	23/1.9	Yes	98/8.2	No	No	No
2	Female	14.2	LCHADD	1.6	No	Negative at first^a1^	0.7	No	–	No	–	Yes	69/5.8	No	No	No
3	Male	9.1	LCHADD	0.3	No	Positive	0.6	Yes	9/0.7	Yes	9/0.7	Yes	78/6.5	No	No	No
4	Female	4.8	LCHADD	0.4	Yes	Positive	1.0	Yes	2/0.2	Yes	3/0.3	Yes	24/2.0	No	No	No
5	Female	5.0	LCHADD	0.0	No	Positive	1.0	No	–	No	–	No	–	No	No	No
6	Female	3.1	VLCADD	0.0	No	Positive	0.0	No	–	Yes	10/0.8	No	–	No	No	No
7	Female	4.1	LCHADD	0.0	No	Positive	0.1	No	–	No	–	No	–	No	No	No
8	Female	4.1	LCHADD	0.0	No	Positive	0.1	No	–	No	–	No	–	No	No	No
9	Male	9.1	CPTII	4.1	No	Positive	7.9	Yes	2/0.2	Yes	3/0.3	No	–	No	No	No
10	Male	11.6	LCHADD	5.5	Yes	Negative at first^a1^	3.7	Yes	5/0.4	Yes	5/0.4	Yes	38/3.2	Yes	No	No
11	Male	13.7	LCHADD	0.0	Yes	Positive	11.6	Yes	113/9.4	Yes	139/11.6^c^	Yes	38/3.2	No	Yes	Yes^e^
12	Male	32.0	VLCADD	36.0	Yes	Not yet established^a^	29.4	Yes	376/31.3	Yes	36/3^c^	No	–	No	No	No
Median		9.1		0.35			1.0		9/0.7		9.5/0.8		53/4.4			

Patients 3, 7 and 8 are siblings; Patient 11 has two affected siblings without triheptanoin therapy; Patient 12 has two affected siblings, one deceased and one without triheptanoin therapy. All other patients have no affected siblings

Patient 1 was previously reported in: JIMD Reports (2014), Karall et al. (https://doi.org/10.1007/8904_2014_313) [14]

Patients 1, 2, 3 and 10 were previously reported in: Orphanet J. Rare Dis. (2015) 10:21, Karall et al. (https://doi.org/10.1186/s13023-015-0236-7) [15], Patient 1 is Patient 2, Patient 2 is Patient 4, Patient 3 is Patient 6, Patient 10 is Patient 8

Patient 10 was previously reported in: Orphanet J Rare Dis. (2018) 13: 122, Lotz-Havla et. al. (https://doi.org/10.1186/s13023-018-0875-6) [16], Patient 10 is Patient 3

C7: triheptanoin; LC-FAOD: long-chain fatty acid oxidation disorder; LCHADD: long-chain 3-hydroxy acyl-CoA dehydrogenase deficiency; VLCADD: very long-chain acyl-CoA dehydrogenase deficiency; CPT2: carnitine palmitoyl transferase 2 deficiency; NG: nasogastric; PEG: percutaneous endoscopic gastrostomy

^a^born before April 2002, when newborn screening for LC-FAOD was implemented in Austria

^a1^Newborn screening was false-negative due to prematurity and parenteral nutrition with carnitine supplementation

^b^At study date, all patients have normal liver function tests

^c^In Patients 1, 6, 10, 11, 12, cardiomyopathy was dilatative; in Patients 3, 4, 9 hypertrophic. At study date Patients 1, 3, 4, 6, 9, 10 have normal cardiac function. In Patients 11 and 12 cardiac function is impaired, cardiomyopathy has not resolved

^d^Retinopathy findings are stable in all patients at study date. Patients 1 and 10 wear glasses for myopia

^e^Wheelchair-bound

In nine patients the diagnosis was established through newborn screening and confirmed by molecular and/or enzymatic testing. Three patients (Patients 1, 10, 12) were detected clinically with metabolic acidosis, hepatopathy, cardiomyopathy and rhabdomyolysis. Patients 1 and 12 were born before the inclusion of LC-FAOD in the Austrian newborn screening panel.

Two patients have very long-chain acyl-CoA dehydrogenase deficiency (VLCADD) (Patients 6 and 12), one carnitine palmitoyltransferase 2 deficiency (CPT 2 deficiency) (Patient 9), the other nine have long-chain 3-hydroxyacyl-CoA dehydrogenase deficiency (LCHADD). Six patients are male, six female. Patients come from ten families, including three siblings from one family of non-consanguineous parents (Patients 3, 7, 8).

At age 3 years, Patient 12 (currently 32 years old) was diagnosed with VLCADD due to cardiomyopathy. Due to worsening cardiac function, the patient was initiated on triheptanoin at age 29 years.

#### Triheptanoin compassionate use program

The hypothesis on the beneficial anaplerotic effect of triheptanoin was first reported in 2002 by Roe et al. [10]. After suffering from repeated episodes of rhabdomyolysis, Patient 1 was started on compassionate use triheptanoin at age 4 years in 2004 [14, 15]. Initially, triheptanoin was provided as an anticorrosive industrial oil by Company Sasol. After good experience with Patient 1, Patients 2, 3 and 10 were also started on triheptanoin. Since 2013, Ultragenyx Pharmaceuticals has provided triheptanoin in a compassionate use program. Patients 4, 5, 6, 7, 8 and 12 joined the compassionate use program in 2016 and 2017, Patients 9 and 11 followed in 2018 and 2019.

### Analysis

#### Statistics focused on descriptive analyses.

The annualized rate of total days of hospitalization (doht) in pre- and post-treatment with triheptanoin was assessed as follows:

$$doht=\frac{Total number of days in hospital}{(Duration of data collection period in days/365.25)}$$

The annualized rate for days in hospital in the one-year pre- and the one-year post-treatment with triheptanoin was calculated as:

$$doh1y=\frac{total number of days in hospital}{365.25}$$

## Results

### Triheptanoin administration

Triheptanoin was started shortly after birth in Patients 6, 7 and 8. The other nine patients were started on triheptanoin between 0.6 and 29.4 (median 3.7, mean 6.7) years of age. Duration between diagnosis and start of triheptanoin was between 0.6 and 26.4 (median 2.9, mean 6.1) years. Median duration of triheptanoin intake was 3.9 (mean 5.3) years, ranging from 1.2 to 15.7 years (Table 2).

**Table 2:** Summary of dietary treatment including tripheptanoin (C7) in 12 Austrian LC-FAOD patients

Patient	Age at start of C7 therapy (years)	Total duration of C7 therapy (years)	Reported adverese events	Triheptanoin (C7) intake		Middle chain fat		Total amount fat including C7 [%]	Daily calorie intake (kcal)	Late evening meals or night feeds		Current weight		Current height		Current BMI	
				g/day	g/kg/ day	C7 in diet [%] of total calories	MCT in diet [%]of total calories			Approx. time	Intake of	kg	percentile	cm	percentile	kg/m^2^	percentile
1	4.8	15.7	None	40	0.50	11.0	0	20	2200	22:00	Late meal^e^	79.6	88	185	76	23.1	50
2	0.7	13.5	None	30	0.57	7.5	7.5	30	2200	22:00	Late meal	52.0	70	159	45	20.5	60
3	0.6	8.5	None	15	0.48	7.5	7.5	30	1900		No	31.0	50	148	97	14.2	10
4	1.0	3.8	None	14	0.80	7.5	7.5	30	1500	22:00	200 ml MCT based formula	17.4	50	111	77	14.1	20
5	1.0	4.0	None	40	1.00	7.5	7.5	30	1500	22:00	250 ml skimmed 0.1% milk	18.4	60	115	96	13.8	13
6	0.0	3.2	None	10	0.59	15.0	10.0	30	1000	22:00	200 ml MCT based formula	16.8	90	100	67	16.9	80
7	0.1	4.0	None	10	0.47	7.5	7.5	30	1000		No	21.0	99	116	99	15.6	50
8	0.1	4.0	None	10	0.58	7.5	7.5	30	1000		No	17.0	60	109	90	14.3	20
9	7.9	1.2^a^	Abdominal pain^a^	20	0.52	13.0	8.0	30	1900	22:00	Late meal	38.0	99	131	30	22.0	95
10	3.7	1.3^b^	Abdominal pain^b^	0^b^	1.00	0.0	30.0	40	2000	03:00	maltodextrin and MCT based formula	37.5	40	141	11	18.9	60
11	11.6	2.1	None	80	1.30	30	0.0	45	2200	03:00	70 ml MCT based formula	62.0	70	163	30	23.3	80
12	29.4	2.2^c^	Abdominal pain^c^	60	0.70	20	0.0	up to 30	2520	22:00	Late meal and 80 g uncooked corn starch	84.0	82^d^	180	55^d^	26.0	89^d^
median	1.0	3.9			0.58	7.5	7.5	30				60	77		50		

Patients 1, 2, 3 and 10 were previously reported: In: Orphanet J Rare Dis. (2015) 10:21, Karall et al. (https://doi.org/10.1186/s13023-015-0236-7), Patient 1 is Patient 2, Patient 2 is Patient 4, Patient 3 is Patient 6, Patient 10 is Patient 8

Patient 1 was previously reported in JIMD Reports (2014), Karall et al. (https://doi.org/10.1007/8904_2014_313

Patient 10 was previously reported in: Orphanet J Rare Dis. (2018) 13: 122, Lotz-Havla et. al. (https://doi.org/10.1186/s13023-018-0875-6), Patient 10 is Patient 3

^a^Patient 9 discontinued C7 therapy (1 g/kg/day) for 1.5 months due to abdominal pain and recommenced at a reduced dosage (0.55 g/kg/day) with no further side-effects reported

^b^In 07/2013, at the age of 5 years, Patient 10 discontinued therapy after 15 months due to abdominal pain;

^c^Patient 12 discontinued C7 therapy (1.2 g/kg/day) for seven months due to abdominal pain and recommenced at a reduced dosage (0.7 g/kg/day) with no further side-effects reported

^d^Adult patient, percentiles at age 18; Kromeyer-Hauschild [13] and Zürcher Longitudinalstudien (1955–2009) [12]

^e^Patient 1 together with the late meal takes: 30 g pegylated corn starch and 200 ml = 300 kcal drink with hydrolyzed protein and fiber, but fat, lactose and gluten free [14]

Nine patients had an uninterrupted intake of triheptanoin and reported no side effects, 11/12 patients have an ongoing intake of triheptanoin on study date (Table 2).

### Adverse events of triheptanoin (Table 2)

Patient 9 was started at 1 g/kg/day and after nine months discontinued therapy for 1.5 months because of abdominal pain. Therapy was recommenced at a lower adjusted dosage of 0.55 g/kg/day. He has taken triheptanoin in that dose for six months and reports no further adverse events.

Patient 10 discontinued triheptanoin at a dosage of 1 g/kg/day after 1.3 years due to abdominal pain and vomiting. No restart was attempted. Patient 10 was previously reported as Patient 3 in Lotz-Havla et al. [16] and in Karall et al. as Patient 8 [15].

Patient 12 discontinued triheptanoin at a dosage of 1.2 g/kg/day after five months due to abdominal pain. He recommenced therapy after eight months at a lower adjusted dosage of 0.7 g/kg/day. He has been on triheptanoin continuously for 24 months and no further adverse events were reported.

Treatment with triheptanoin was not associated with excessive weight gain. Eleven patients show adequate BMI between percentile 10 and 89 (percentile mean 44, median 50). Patient 9 has a BMI of 22 kg/m^2^ (percentile 95), probably due to eating habits.

All patients are on a fat-defined, high-carbohydrate diet with an age-appropriate protein and caloric intake (Table 2). Of the total caloric intake, median fat intake was 30% (range 20–45%), 7.5% (range 0–30%) thereof is median medium-chain triglyceride (MCT) intake. In addition, for supplementation of long-chain essential fatty acids all patients receive walnut-oil (median 0.27 g/kg/day, range 0.06–0.48 g/kg/day). The median amount of triheptanoin intake is 0.58 (range 0.48–1.30) g/kg/day, equaling a median total daily calorie intake of 7.5% (ranging from 7.5% to 30%).

### Triheptanoin treatment decreases hospitalizations per year (Fig. 2)

**Fig. 2:**
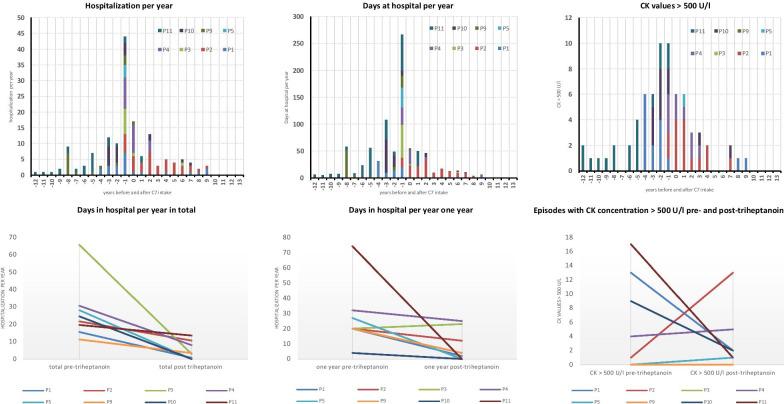
Outcome: Frequency and duration of hospitalizations and creatine kinase (CK) concentrations before and after triheptanoin (C7) treatment in eight Austrian patients with LC-FAOD. Each patient is shown by a different color. The outcome parameters are normalized to the respective commencement of triheptanoin (point 0: start of C7). The years before C7 start are seen to the left of point 0 on the x axis and therefore negative. Panels 1a–3a show parameters per year, Panels 1b–3b show the total sum of the parameters before vs. after C7 therapy. P1: Patient 1, P2: Patient 2, etc.

As a parameter of metabolic stability, we compared total days of hospitalization (doht) pre- and post triheptanoin as well as days of hospitalization (doh1y) one year prior to one year after start of therapy with triheptanoin. We excluded four patients from these calculations: In Patients 6, 7 and 8 time interval between birth and commencement of triheptanoin intake was less than six months, and in Patient 12, in whom triheptanoin was started at age 29 years to treat cardiac disease, the retrieved data were incomplete.

For the 8/12 included patients, the total days of hospitalization per year (doht) decreased by 82.3% from mean 27.1 (median 23.1, range 11–65) days/year during the pre-triheptanoin period (mean 3.9, median 2.3, range 0.61–11.64 years) to mean 4.8 (median 3.1, range 0–13) days/year during the post-triheptanoin period (mean 7, median 5.9, range 1.18–15.72 years) (Fig. 2, Panels 1a and 1b).

Days of hospitalization in the year pre-triheptanoin (doh1y) as compared to the year post-triheptanoin decreased by 69.75% from mean 27.1 (median 20, range 4–74) days/year to mean 8.2 (median 3, range 0–25) days/year (Fig. 2, Panels 2a and 2b).

### Clinical signs, short- and long-term symptoms and complications

All patients are in stable clinical condition to date with BMI percentiles between P10 and P95 (Table 2).

Hypoglycemia was present in Patients 1, 2, 4, 10, 11 and 12 at the time of diagnosis and before start of triheptanoin. Only in Patient 4, where LCHADD was diagnosed in the first week of life through newborn screening, episodes of hypoglycemia occurred until the age of 3 years, due to poor adherence to dietary regimen, but resolved after frequency of meals and caloric intake was adapted to be adequate to age. In Patient 4, triheptanoin was started at the age of 12 months (see Table 1).

In summary seven patients developed hepatopathy between 0.2 and 31.3 (mean 6.3, median 0.7) years of age, defined as elevated liver enzymes (GOT, GPT, GGT) and sonographic findings (Table 1). At date, all patients have normal liver function.

Eight patients developed cardiomyopathy, defined as fraction shortening (FS) < 25% and/or ejection fraction (EF) < 50% in at least one echocardiographic screening between 0.3 and 11.6 (mean 2.4, median 0.8) years of age (Table 1). Five patients showed a dilative, three a hypertrophic cardiomyopathy, seven were on medication. At last echocardiography, six showed normal cardiac function; in Patients 11 and 12, cardiomyopathy had not completely resolved (Table 1).

Retinopathy has been reported in 6/9 LCHADD patients, first diagnosed between 2.0 and 8.2 (mean 4.7, median 4.4) years of age. Patients 1 and 10 show impaired vision due to myopia and wear glasses (Table 1). Patient 11 developed polyneuropathy, firstly diagnosed around age 10 years, and slowly progressed over time. In his apartment he does not need a walking aid but for longer distances he is dependent on a wheelchair (Table 1). Triheptanoin, which was started at age 11.6 years, has not had an obvious impact on the polyneuropathy, but observation time is still short.

As part of clinical follow-up checks, patients were continuously clinically and neurologically examined and any restrictions in daily life activities were raised. Collectively, our patients report adequate to good school performance, except Patient 11 who has needed school support since the age of 12 years. Patient 12 is employed and lives independently. All 12 patients lead an independent life and report no subjective restrictions in daily life.

### Triheptanoin decreases episodes with elevated CK followed by hospitalization

Elevated creatine kinase (CK), the marker of rhabdomyolysis/muscle involvement (defined as CK over 500 U/l followed by hospitalization), was recorded in 75 episodes in 8/12 patients. Three patients never showed CK concentrations > 500 U/l. For Patient 12 no data are available.

CK concentrations ranged from 500 U/l to 142.700 U/l (median 4.360 U/l; mean 12.630 U/l). The range of episodes with a CK > 500 U/l is one to 18 (median 10) episodes per patient.

Comparing CK concentrations > 500 U/l pre- and post-triheptanoin, we calculated a mean reduction of 45% in total episodes pre- vs post triheptanoin treatment. We recorded a total of 13 episodes per patient year (44 episodes in 3.4 patient years) before triheptanoin (mean 5.5, median 2.5, ranging from 0 to 17 episodes/patient) and a total of 3.5 episodes per patient year (24 episodes in 6.7 patient years) after commencement of triheptanoin (mean 3, median 1.5, ranging from 1 to 13 episodes/patient) (Fig. 2, Panels 3a and 3b).

### Dietary long-term management

In our patient cohort, 11 patients eat self-sufficiently. Patient 10 is regularly fed via PEG. Since age 3 months he has suffered frequent vomiting and refused to eat. At age 1 year, he received a PEG tube. At date, he increasingly shows interest in food, feeding via PEG is performed only during the night. Seven (Patients 1, 2, 4, 5, 6, 9, 12) have a late evening meal around 22:00 pm (Table 2), three (Patients 3, 7, 8) have no late meals or night feeds. Patients 10 and 11 have night feeds (around 02:00–03:00 am), Patient 10 via PEG and Patient 11 orally (Table 2).

## Discussion

Besides establishing an early diagnosis, the challenge in LC-FAOD management is to determine the adequate amount of fat intake, maintain an anabolic state, and tamper metabolic crises. For about 20 years, additional intake of odd-middle-chain fatty acids, i.e. triheptanoin, has played an important role [10], even though its mode of action at a cellular level is still under investigation [17]. However, even though triheptanoin treatment renders promising results in clinical practice [14, 15], the challenge to quantify its effect in patients remains as reliable biochemical markers are lacking. Thus, surrogate markers are chosen to show beneficial effects of triheptanoin, like hospitalization needed before and after initiation of treatment.

In this paper we summarize our long-term experience with triheptanoin treatment in 12 Austrian patients with LC-FAOD (Tables 1 and 2; Fig. 2).

We show that before and after triheptanoin treatment the total days of hospitalization are reduced by 82.3%, and when considering only the one year before and the one year after treatment by 69.75% (Fig. 2). In addition, the creatine kinase concentrations above 500 U/l also decreased (Fig. 2, Panels 3a and 3b). Similar observations (reduction in episodes of rhabdomyolysis, reduction in hospitalizations per year) have been reported [2, 6, 7].

Our approach to triheptanoin therapy resulted from a first very positive experience with the oil in Patient 1 over 15 years ago [14, 15]. As the initial preparation was an industrial oil that was used as an anticorrosive and was not very palatable and quite aggressive to plastic materials, after trying it ourselves and with informed consent of the parents of Patient 1 we started with an initial dosing of 0.5 g/kg/day. The rest of the LC-FAOD dietary treatment remained the same, i.e. 2 g/kg/day MCT oil (equaling 10% of total energy intake from MCT fat), total fat intake around 30% of total energy intake and careful substitution of essential long-chain fatty acids with walnut oil (about 4% of total energy intake). The FDA recommends replacing MCT oil with triheptanoin. However, our patients show that parallel administration of MCT oil and triheptanoin is well possible. Thus, patients profit from the positive effects of triheptanoin with lower dosage and thus less gastrointestinal side effects. MCT oil can then also be used for cooking and meal preparation. As this regimen was seen to be successful, we applied it—adapted for age—to all subsequent patients. Thus, in our cohort only Patients 11 and 12 have a total substitution of MCT by triheptanoin, all the others have a mixture of MCT and triheptanoin intake (Table 2).

In other studies [2, 6, 7, 9, 18], triheptanoin intake was higher (25–35% of total calories, as tolerated, which is equivalent to approximately 2–4 g/kg in infants and young children), in our opinion leaving less space for intake of standard diet products. Therefore, we try to avoid a too stringent fat restriction, as over time that will inevitably lead to a situation in which patients are prone to catabolism because fat is needed in adequate quantities for growth and development of e.g. membranes. In our treatment philosophy, we do not limit our patients' total fat intake too strictly when they are well, but implement long-chain fat restriction alongside carbohydrate-based caloric intake in emergency regimens. In our cohort, the median fat intake from total energy is 30%. In summary, we assume that together with the beneficial effects of triheptanoin, intake of enough fat leads to greater metabolic stability and better overall outcome.

Moreover, a higher dosage of triheptanoin might cause more adverse events: Three patients (Patients 9, 10, 12) were started on a higher triheptanoin dosage (mean 1.1 g/kg/day), which led to discontinuation of triheptanoin due to abdominal pain and diarrhea. After dosage reduction to 0.7 and 0.5 g/kg/day, Patient 9 and 12, respectively, had no further adverse events; Patient 10 never recommenced triheptanoin therapy (Table 2). All other patients have tolerated triheptanoin well and without problems (Table 2). We attribute the good tolerability to the smaller amount of triheptanoin prescribed (on average 0.58 g/kg/day, equaling 7.5% of total daily caloric intake) in comparison to other groups.

The current study is limited by its uncontrolled, open-label, retrospective design. In addition, the cohort is relatively small and heterogeneous in terms of age, disease presentation and severity. However, the study strength is its long observation period and the rather homogeneous treatment regimen. We analyzed and recorded all assessments pre- and post-triheptanoin in the same manner to eliminate assessment or detection bias. Furthermore, we can deduce that clinical events resulting in hospitalization would have been more frequently reported and carefully monitored because of the intake of triheptanoin as a compassionate use drug.

Most important issue: As the younger age in children is associated with more frequent infections and the decrease in hospitalization days could have been due to the increase in age alone, we normalized the evaluation time to the start of the triheptanoin treatment. Thus, the chosen parameter “hospitalization days one year before compared to one year after commencement of triheptanoin” is fairly independent of the increasing age of the individual patient. It is very unlikely that increase in age alone would result in such a significant reduction in these parameters.

In summary, our data show that the outcome of LC-FAODs under treatment with triheptanoin is favorable and safe in long-term observation. We consider it essential to keep patients with LC-FAOD in an anabolic state, with a dietary regimen that allows daily life to be as normal as possible.

## Conclusions

Daily treatment with 0.5–1.0 g/kg/day triheptanoin while allowing a total fat intake of up to 30% of total daily energy shows good long-term clinical outcome in patients with LC-FAOD. Maintaining patients in an anabolic state is crucial and outweighs the effect of stringent fat restriction.

## Data Availability

The datasets used and/or analysed during the current study are available from the corresponding author on reasonable request.

## Abbreviations

LC-FAOD: Long-chain fatty acid oxidation disorders
LCHADD: Long-chain 3-hydroxyacyl-CoA dehydrogenase deficiency
VLCADD: Very long-chain acyl-CoA dehydrogenase deficiency
CPT 2: Carnitine palmitoyltransferase 2 deficiency
CK: Creatine kinase
BMI: Body mass index
MCT: Medium-chain triglycerides
FS: Fraction of shortening
EF: Ejection fraction
